# Study on the Biological Characteristics of Dark Septate Endophytes under Drought and Cadmium Stress and Their Effects on Regulating the Stress Resistance of *Astragalus membranaceus*

**DOI:** 10.3390/jof10070491

**Published:** 2024-07-16

**Authors:** Duo Wang, Yali Xie, Wanyi Zhang, Li Yao, Chao He, Xueli He

**Affiliations:** 1 School of Life Sciences, Hebei University, Baoding 071002, China; wd18740746653@163.com (D.W.); xieyali998@163.com (Y.X.); zwaner041117@126.com (W.Z.); yaoli202202@163.com (L.Y.); 2 Institute of Medicinal Plant Development, Chinese Academy of Medical Sciences & Peking Union Medical College, Beijing 100193, China

**Keywords:** dark septate endophyte, performance, tolerance stress, *Astragalus membranaceus*, drought stress, cadmium pollution

## Abstract

*Astragalus membranaceus* is a famous traditional medicinal plant. However, drought and cadmium (Cd) pollution are the main abiotic stress factors that affect plant growth and yield and the ability to improve the host’s stress resistance through the use of beneficial endophytic fungi. To evaluate the tolerance of dark septate endophytes (DSE) to various abiotic stresses, 10 DSE strains [*Microsphaeropsis cytisi* (*Mc*), *Alternaria alstroemeriae* (*Aa*), *Stagonosporopsis lupini* (*Sl*), *Neocamarosporium phragmitis* (*Np*), *Paraphoma chlamydocopiosa* (*Pc*), *Macrophomina phaseolina* (*Mp’*), *Papulaspora equi* (*Pe*), *Alternaria tellustris* (*At*), *Macrophomina pseudophaseolina* (*Mp*), and *Paraphoma radicina* (*Pr*)] were investigated under different drought and Cd stressors in vitro by using solid-plate cultures and liquid-shaker cultures in the current study. The experiments involved using varying concentrations of PEG (0, 9, 18, and 27%) and Cd^2+^ (0, 25, 50, and 100 mg/L) to simulate different stress conditions on DSE. Additionally, the effect of DSE (*Np* and *At*) on the growth of *A. membranaceus* at different field water capacities (70% and 40%) and at different CdCl_2_ concentrations (0, 5, 10, and 15 mg Cd/kg) in soil was studied. The results demonstrated that the colony growth rates of *Aa*, *Np*, *Pc*, *Mp’*, and *Mp* were the first to reach the maximum diameter at a PEG concentration of 18%. *Aa*, *Np*, and *At* remained growth-active at 100 mg Cd/L. In addition, *Aa*, *Np*, and *At* were selected for drought and Cd stress tests. The results of the drought-combined-with-Cd-stress solid culture indicated that the growth rate of *Np* was significantly superior to that of the other strains. In the liquid culture condition, the biomasses of *Np* and *Aa* were the highest, with biomasses of 1.39 g and 1.23 g under the concentration of 18% + 25 mg Cd/L, and *At* had the highest biomass of 1.71 g at 18% + 50 mg Cd/L concentration, respectively. The CAT and POD activities of *Np* reached their peak levels at concentrations of 27% + 50 mg Cd/L and 27% + 25 mg Cd/L, respectively. Compared to the control, these levels indicated increases of 416.97% and 573.12%, respectively. *Aa*, *Np*, and *At* positively influenced SOD activity. The glutathione (GSH) contents of *Aa*, *Np*, and *At* were increased under different combined stressors of drought and Cd. The structural-equation-modeling (SEM) analysis revealed that *Aa* positively influenced biomass and negatively affected Cd content, while *Np* and *At* positively influenced Cd content. Under the stress of 40% field-water capacity and the synergistic stress of 40% field-water capacity and 5 mg Cd/kg soil, *Np* and *At* significantly increased root weight of *A. membranaceus*. This study provides guidance for the establishment of agricultural planting systems and has good development and utilization value.

## 1. Introduction

Drought and heavy-metal pollution are the most prevalent and detrimental abiotic stressors that significantly impede plant growth and survival [1,2,3]. As a non-essential element for plants, cadmium (Cd) is recognized as one of the most toxic heavy-metal pollutants, with the potential to degrade agricultural product quality, diminish yields, and contaminate the food chain, thereby posing a significant threat to human health. Soil Cd levels can vary widely, particularly in China, ranging from 0.003 to 9.57 mg/kg [3]. The compounding effects of drought and Cd stress, which are frequently encountered in many regions, further amplify the challenges for plant life [4,5]. Such dual stressors can lead to nutritional imbalances, a reduction in chlorophyll synthesis, and the disruption of enzymatic activities and metabolic functions, all of which severely impact plant growth and agricultural productivity. It is evident that the study of single stress factors is insufficient to address the demands of sustainable agricultural development, necessitating a comprehensive understanding of the synergistic effects of multiple abiotic stressors.

*Astragalus membranaceus*, a renowned traditional medicinal plant, is celebrated for its dried roots, which are rich in bioactive compounds, including flavonoids, saponins, and polysaccharides. These constituents are known to mitigate the effects of hyperglycemia, exhibit anti-aging properties, and possess anti-tumor activities [6]. Beyond its medicinal significance, *A. membranaceus* demonstrates resilience to harsh environmental conditions such as drought and cold, contributing to ecological benefits through its windbreak and sand-fixation capabilities, thereby enhancing the ecological environment. However, the prevalence of concurrent drought and Cd contamination in various regions poses a significant challenge, necessitating research into the plant’s adaptability and potential remediation strategies in the face of these dual stressors. Research reports have underscored a concerning issue regarding the quality of Chinese medicinal materials, revealing that eight types, including *A. membranaceus*, have been identified as having Cd content exceeding permissible levels in 27 provinces and regions across China, with an alarming over-standard rate of 17.73%—1.4 times the established Cd limit standard. This issue is particularly pronounced in Northwest China, characterized by low precipitation and high evaporation rates, where as much as 20.00% of *A. membranaceus* samples from Shaanxi province have been found to exceed the Cd safety threshold [7]. Addressing the enhancement of plant tolerance to the combined stressors of drought and Cd contamination is thus an urgent challenge in the field. Studies have suggested that the introduction of beneficial microbes [8], such as dark septate endophytes (DSE), may offer a promising avenue for improving plant stress resilience and overall growth, warranting further investigation for sustainable agricultural practices and medicinal plant cultivation.

DSEs constitute a diverse group of ascomycetes fungi known for their ability to colonize the roots of living plants without inducing any apparent detrimental effects. Characterized by the presence of microsclerotia and pigmented hyphae, these endophytic fungi are ubiquitous in nearly all natural ecosystems, particularly thriving under challenging conditions such as those found in saline, polluted, and arid environments [9]. Empirical research has demonstrated that DSEs can significantly enhance the host plants’ water absorption capacity, promote growth, and bolster their stress tolerance [10,11]. Under drought conditions, DSEs can form a complex and continuous network within the plant’s root system to improve underground and above-ground water transportation and enhance the drought resistance of plants [12]. Under heavy-metal pollution conditions, DSEs hinder the migration of heavy-metal ions in plants by their adsorption abilities and improve plant tolerance to heavy-metal stress [13,14].

DSEs have currently been reported to play a role in modulating the physiological and biochemical stress responses in plants, but the existing literature primarily focuses on their effects under single-stress conditions. For example, under drought stress, DSEs have been shown to augment the biomass and active compound levels in *Isatis indigotica*, as well as to positively influence the morphology, biomass, physiological traits, and bioactive content of licorice plants. The inoculation of DSEs in wheat under drought conditions not only promotes growth but also diminishes the water consumption of seedlings, thereby facilitating water-efficient agricultural practices [15,16,17]. In the context of heavy-metal stress, DSEs have been demonstrated to significantly increase the biomass and height of maize plants exposed to Cd, with higher Cd sequestration in the root cell walls of DSE-inoculated plants, indicating an enhanced tolerance to Cd. The underlying mechanism involves alterations in root morphology and the facilitation of Cd binding to cell walls and phosphates. Furthermore, DSEs have been reported to enhance Cd tolerance in rice, reducing its accumulation in the root system and translocation to the stem. This process is associated with the upregulation of the SNARE Syntaxin 1 gene, which is implicated in the regulation and alleviation of Cd accumulation by DSEs [18,19,20].

However, plants are often subjected to the combined stresses of drought and Cd pollution. The screening of DSE strains with combined resistance to drought and Cd is not only crucial for application in phytoremediation but also for the regulation of plant stress resistance. At present, there have been few studies on DSE-resistant strains; however, the screening and application of DSE-resistant strains will be the main research direction and goal in the future [8,21]. To elucidate the mechanisms underlying the tolerance of DSEs to synergistic environmental stresses, this study designed in vitro assays incorporating both single-factor and compound-factor conditions, utilizing varying concentrations of PEG-6000 to simulate osmotic stress and a range of Cd concentrations to simulate heavy-metal stress. These assays were conducted to assess the influence of combined stressors on the growth performance of ten distinct DSE strains. Concurrently, field experiments were established to examine the impact of DSE under different soil water capacities and Cd levels on the growth of *A. membranaceus*. The objectives of this study encompass the exploration of the following topics: (1) How does DSE tolerate drought stress in vitro? (2) How does DSE tolerate Cd stress in vitro? (3) How does DSE tolerate combined drought and Cd stress in vitro? (4) What is the effect of DSE on the growth of *A. membranaceus* under drought and Cd single and synergistic stresses?

## 2. Materials and Methods

### 2.1. Fungal Materials of DSE

In this study, 10 DSE strains were isolated from the roots of different plants (Table 1) and isolated from the fine roots of *Glycyrrhiza uralensis*, *Isatis indigotica*, *Astragalus membranaceus*, *Lycium ruthenicum*, *Dendranthema morifolium*, and *Salvia miltiorrhiza* [22,23,24,25,26,27].

Root segments of these plants were selected for surface disinfection. They were rinsed several times with sterile water, sterilized with 75% ethanol for 5 min, sterilized with sodium hypochlorite for 5 min, rinsed several times with sterile water, and dried on sterile filter paper. The root segments were cultured in potato-dextrose-agar medium (PDA medium, selected from Beijing Aoboxing Bio-Technology Co., Ltd., Beijing, China) with antibiotic supplements (ampicillin and streptomycin sulfate) in Petri dishes. Sterilized root samples were incubated in the dark at 27 °C and observed daily. When black mycelia grew around the surface of the root segment, the growth condition was good. Fresh mycelia were selected from a super-clean worktable, placed in a new PDA medium for purification, and then cultured in the dark at 27 °C. Fresh mycelium (approximately 50 mg) was scraped from each colony, and colony DNA was extracted with a genomic DNA isolation kit (Beijing Suolaibao Technology Co., Ltd., SolarBio, Beijing, China). The primers ITS4 (5′-TCCTCCGCTTATTGATATGC-3′) and ITS5 (5′-GGAAGTAAAAGTCGTAACAAGG-3′) were applied to amplify the colony DNA, and the amplified products were sequenced. The BLAST tool in NCBI (https://www.ncbi.nlm.nih.gov/, 2 May 2024) was used for comparison, and a phylogenetic tree was constructed using the MEGA version 6.0 software [28]. The ITS1-5.8S-ITS2 ribosomal DNA sequences of these DSE strains were uploaded to GenBank with the following specific accession numbers, and their names were *Microsphaeropsis cytisi* (*Mc*), *Alternaria alstroemeriae* (*Aa*), *Stagonosporopsis lupini* (*Sl*), *Neocamarosporium phragmitis* (*Np*), *Paraphoma chlamydocopiosa* (*Pc*), *Macrophomina phaseolina* (*Mp’*), *Papulaspora equi* (*Pe*), *Alternaria tellustris* (*At*), *Macrophomina pseudophaseolina* (*Mp*), and *Paraphoma radicina* (*Pr*), respectively. The strains were stored at 4 °C in the Mycorrhizal Biology Laboratory of Hebei University.

### 2.2. Growth Conditions

The seeds of *A. membranaceus* were collected from the Gansu Province of China and stored at 4 °C. Uniform and full *A. membranaceus* seeds were selected and rinsed three times with distilled water. The seeds were soaked in distilled water for 12 h, placed in a seedling tray with a small amount of distilled water, then placed in an incubator at 25 °C under shade and set aside. The seeds germinated in incubators for one week before cultivation. The growth substrate used was a mixture of 1:2 (*W*:*W*) sand (less than 2 mm) and soil. A completely randomized three-factor (three DSE inoculation treatments × two drought stress treatments × four Cd concentrations) block group experimental design was used. Factor 1 was the DSE strain, where the inoculation treatments included two distinct strains, designated as *Np* and *At*, alongside a blank control group that received no DSE inoculation. Factor 2 was the soil moisture treatment. One simulating normal moisture levels was set at 70% field water capacity, and the other induced drought stress at 40% field water capacity. These treatments were designed to evaluate the plants’ responses to varying water availabilities. Additionally, Factor 3 introduced a soil Cd stress treatment, where we prepared solutions of cadmium chloride (CdCl_2_) at concentrations of 67.5 mg/L, 135 mg/L, and 202.5 mg/L and added 100 mL of each to the soil substrate to create a gradient of Cd solution, and the blank control group was added with an equal amount of distilled water. Four levels of Cd stress were set at 0, 5, 10, and 15 mg Cd/kg, respectively. Four replicates were set up for each treatment group, with a total of 96 pots. Different single and synergistic stress-treatment groups were established (Table 2).

A total of 800 g of mixed substrate was weighed and placed in plastic pots (mouth diameter of 13 m, bottom diameter of 10 m, height of 12 cm); a 7 mm diameter hole punch was used to intercept the fungus cake from the PDA medium, and four pieces were placed on the top layer of the substrate in each pot and finally covered with 550 g of mixed substrate. Four pieces of blank PDA medium were taken in the same way as the DSE blank control group [29]. Four well-grown and uniform *A. membranaceus* seedlings were selected for planting in each pot, and after planting, all potted plants were placed in an artificial climate incubator with a photoperiod of 14/10 h, 24/22 °C (day/night) and an average relative humidity of 60%. The experiment started on 25 February 2024 and concluded with the harvest on 25 June 2024 for a period of 4 months. After 45 d, *A. membranaceus* was treated with various stress treatments. During the experimental period, water loss was regularly replenished with distilled water, and soil moisture was maintained by regular weighing. The position of the seedling pots was randomly changed weekly to ensure that they were not affected by positional effects. The height of each plant before harvest was recorded. Above-ground plants and underground roots were harvested separately. The roots were rinsed in tap water and then three times in distilled water, drained, and weighed.

### 2.3. Drought Tolerance of DSE Strains

A 120 mL PDA medium was prepared and amended with polyethylene glycol (PEG-6000) and phytagel to imitate a controlled osmotic-stress environment. The ratio of PEG-6000 to phytagel was maintained at 17:1, with incremental additions of 0, 11.87, 26.34, and 44.38 g of PEG-6000 to establish a range of osmotic pressures, corresponding to PEG mass fractions of 0%, 9%, 18%, and 27% [30]. The medium was heated and agitated to ensure the uniform distribution of PEG-6000. Following a 14-day cultivation period, a 5 mm diameter inoculum of DSE colonies was extracted from the peripheral region and placed at the center of the PDA medium with varying drought-stress gradients. Subsequently, the inoculated plates were incubated at 27 °C for 14 d in a dark, inverted incubator. Each treatment was replicated three times to ensure experimental rigor.

### 2.4. Cd Tolerance of DSE Strains

The CdCl_2_·2.5H_2_O was prepared with a Cd ion concentration of 10 mg/mL. Each 250 mL conical bottle was filled with 100 mL PDA medium, and 10 mg/mL of Cd ion master batch was added at 0, 0.25, 0.5, and 1 mL successively. The concentration gradients of Cd ion were set as 0, 25, 50, and 100 mg/L [31]. After the DSE colonies were cultivated for 14 d, a 5 mm diameter fungal inoculum was taken from their periphery. The medium, adjusted to reflect a spectrum of Cd concentration gradients, was centrally positioned for uniformity. Subsequent to inoculation, the cultures were maintained under a 14-day incubation regimen at 27 °C within a dark, inverted incubator, ensuring consistent and controlled environmental conditions. Each experimental condition was replicated three times.

### 2.5. Drought and Cd Tolerance of DSE Strains

#### 2.5.1. Solid Culture of DSE

Drought-and-Cd-combined stress was simulated by adding PEG-6000 and Cd ion master batch at a concentration of 10 mg/mL to the PDA medium at different ratios, and the combined stress concentration gradients were set to 0, 9% + 25 mg Cd/L, 9% + 50 mg Cd/L, 9% + 100 mg Cd/L, 18% + 25 mg Cd/L, 18% + 50 mg Cd/L, 18% + 100 mg Cd/L, 27% + 25 mg Cd/L, 27% + 50 mg Cd/L, and 27% + 100 mg Cd/L [30]. Colonies of DSE, after 14 d of cultivation, were sampled for a fungal inoculum of 5 mm in diameter from their periphery. These were placed at the center of the PDA medium, which was prepared with varying gradients of drought-and-Cd-combined stress concentrations. The inoculated medium was then incubated at 27 °C for a period of 14 d in a dark, inverted incubator. A control group (CK), representing conventional culture conditions without any additional treatment, was included alongside ten experimental treatments, each with three replicates.

#### 2.5.2. Liquid Culture of DSEs

Each 250 mL conical flask contained 120 mL of Potato Dextrose Water liquid medium (PD liquid medium, sourced from Qingdao Hope Bio-Technology Co., Ltd., Qingdao, China). To this medium, a PEG solid and a 10 mg/mL master batch of Cd ions were incorporated in varying proportions to achieve a consistent level of combined stress as previously described. After the DSE colonies were cultivated for 14 d, three fungal inoculations (5 mm in diameter) were removed from their periphery, inoculated into a PD liquid medium, and incubated on a shaker at a constant temperature (27 °C, 150 r/min) for 14 d. A control group (CK), representing conventional culture conditions without any additional treatment, was included alongside ten experimental treatments, each with three replicates. Following incubation in liquid media, the mycelium and culture liquid were separated using a SHD-III type circulating water multi-purpose vacuum pump. Randomly, two sections of the fresh mycelia were separated. One portion was utilized to measure the levels of melanin, soluble protein, superoxide dismutase (SOD), glutathione (GSH), malondialdehyde (MDA), peroxidase (POD) activity, and catalase (CAT). To determine the Cd content and soluble sugar, when the other portion reached a consistent weight, it was weighed and dried at 80 °C.

### 2.6. DSE Growth and Cd Content

The growth of DSE colonies under various conditions of drought and Cd stress, both individually and in combination, was monitored by employing the cross-measurement technique to determine the colony diameter. This assessment was conducted every 24 h over a 14-day period [32]. The tolerance index (TI) of the DSE strain was calculated by measuring the colony growth after treatment (cm) divided by the control colony growth (cm) [33]. The concentration causing a 50% growth inhibition (IC50) of DSE strains was calculated by the method of Medina-Armijo et al. [31]. On the 8th day of culture, photos were taken to record the colony morphology. The total biomass of DSE strains was obtained by converting the water content ratio of part of the mycelium, and the total biomass = total fresh weight × dry weight/partial fresh weight.

The Cd content of the dry mycelium was determined using an inductively coupled plasma optical emission spectrometer (ICP-OES, Beijing Jianling Technology Co., Ltd., Beijing, China). A grey or white residue was formed by heating the dried hyphae to 550 °C in a crucible that had been weighed. HClO4 (1 mL) was added. The acid treatment process was repeated after heating the hyphae on a heat plate. Concentrated HNO3 in the amount of 1 mL was used to dissolve the remainder, diluted and fixed to 25 mL (grade 1 water) [14].

### 2.7. Determination of DSE Antioxidant Enzyme Activity

Mycelial SOD activity was determined by a nitro-tetrazolium chloride blue-light reduction method [34]. Firstly, 0.2 g of fresh mycelium was weighed using an electronic balance and placed in a 5 mL centrifuge tube. Subsequently, 4 mL of 50 mM phosphate buffer (pH 7.8) was added in an ice bath. The sample was then ground with a high-throughput tissue grinder and centrifuged at 10,000× *g* and 4 °C for 10 min. Then, 0.3 mL of the supernatant was aspirated, and 3.8 mL of a 50 mM phosphate buffer (pH 7.8), 0.3 mL of methionine, 0.3 mL of nitro-tetrazolium blue chloride, and 0.3 mL of riboflavin were added in turn. The light and dark response groups served as controls and were placed in a light incubator under 4000 Lx fluorescent illumination for a duration of 20 min. Subsequently, the absorbance was measured at 560 nm using a spectrophotometer (model 752 N, Shanghai INESA Instrument Analytical Instruments Co., Ltd., Shanghai, China).

Mycelial POD activity was determined by the guaiacol colorimetric method [35], using a change in A470 of 0.01 per minute as one unit (U) of peroxidase activity. Firstly, 0.1 g of fresh mycelium was weighed in a 5 mL centrifuge tube with an electronic balance, and a 5-fold amount of 50 mM phosphate buffer (pH 7.8) was added in an ice bath; then, the sample was ground with a high-throughput tissue grinder, followed by centrifugation at 15,000× *g* and 4 °C for 10 min. Then, 1 mL of 0.3% hydrogen peroxide (H_2_O_2_), 0.95 mL of 0.2% guaiacol, and 1 mL of 50 mM phosphate buffer (pH 7.8) were sequentially added to a 3 mL reaction system. The reaction was initiated by adding 0.05 mL of enzyme solution, followed by shaking well, and the reaction was immediately timed. A blank tube without H_2_O_2_ was used to adjust to zero to measure the absorbance value, and the absorbance value was measured at 470 nm by spectrophotometer.

Mycelial CAT activity was quantified spectrophotometrically by monitoring ultraviolet (UV) absorbance [36]. A decrease of 0.01 in absorbance at 240 nm per minute was defined as the activity unit (U) for catalase. Firstly, 0.1 g of fresh mycelium was weighed in a 5 mL centrifuge tube with an electronic balance, followed by the addition of a 5-fold amount of 50 mM phosphate buffer (pH 7.8) in an ice bath; the sample was ground with a high-throughput tissue grinder, followed by centrifugation at 15,000× *g* and 4 °C for 10 min. The reaction was initiated by the sequential introduction of 1 mL of 0.3% H_2_O_2_, followed by the addition of 1.95 mL water (grade 1) and 0.05 mL of an enzyme solution into a 3 mL reaction system. Upon mixing, the reaction was promptly timed, and the contents were thoroughly agitated to ensure homogeneity. The absorbance value was measured by zero adjustment of a blank tube without H_2_O_2_, and the absorbance value was measured at 240 nm by a spectrophotometer.

### 2.8. Determination of DSE Resistance Parameters

The NaOH extraction method was used to determine the mycelial melanin concentration [37]. Firstly, 0.05 g of fresh mycelium was weighed in 1 mol L^−1^ sodium hydroxide in an electronic balance and heated at 100 °C for 5 h. Then, the cooled mycelium was filtered, and 7 M hydrochloric acid (pH 2.0) was added. Following the precipitate’s extraction, it was washed, 1 mol L^−1^ sodium hydroxide was used to dissolve it, and it was centrifuged at 10,000× *g* for 15 min to determine the amount of melanin extracted [38]. A standard curve was established by spectrophotometer at 459 nm, and melanin content was calculated.

The mycelial-soluble-protein concentration was determined by using the colorimetric method of Thomas Brilliant Blue [39]. Firstly, 0.2 g of fresh mycelium was weighed in a 5 mL centrifuge tube with an electronic balance, then 4 mL of 50 mM phosphate buffer (pH 7.8) was added, and the sample was ground with a high-throughput tissue grinder, followed by centrifugation at 10,000× *g* for 10 min. The supernatant was aspirated for 0.1 mL, 0.9 mL of distilled water was added, 5 mL of Caumas Brilliant Blue was added, and the distilled water was used as a control. Then, the absorbance value at 595 nm was measured with a spectrophotometer.

The concentration of mycelial soluble sugars was measured using the anthrone colorimetric method [40]. Firstly, 0.05 g dry mycelium was weighed in a 5 mL centrifuge tube with an electronic balance, and after grinding, 4 mL of 80% alcohol was added, and the supernatant was transferred to a 10 mL centrifugal tube with a water bath at 80 °C for 30 min, with the above steps repeated. Subsequently, a small amount of activated charcoal was added, and a water bath at 80 °C was performed for 30 min. The sample was diluted with distilled water to a final volume of 10 mL. A 250 μL aliquot was then filtered and transferred into a glass test tube. Subsequently, 5 mL of anthrone reagent was added, and the mixture was subjected to a boiling-water bath for 10 min. Distilled water served as a control in this assay. The absorbance was measured at 625 nm using a spectrophotometer.

The 5,5-dithiobis-(2-nitrobenzoic acid) (DTNB) method was used to measure the concentration of GSH in mycelial [41]. Firstly, 0.2 g of fresh mycelium was weighed in a 5 mL centrifuge tube with an electronic balance, 4 mL of 10% trichloroacetic acid was added, and the sample was ground with a high-throughput tissue grinder, followed by centrifugation at 10,000× *g* for 10 min. Then, 0.25 mL of the supernatant was aspirated in a glass test tube, and 2.6 mL of NaH2PO4 and 0.15 mL of dithiobis-(2-nitrobenzoic acid) were added in order, and then the glass test tube was shaken well. Thereafter, the reaction was kept at 30 °C for 10 min, and the absorbance value was determined using a spectrophotometer at 412 nm.

Mycelial MDA concentration was determined using the thiobarbituric-acid method [42]. Firstly, 0.2 g of fresh mycelium was weighed in a 5 mL centrifuge tube with an electronic balance, 4 mL of 10% trichloroacetic acid was added, and the sample was ground with a high-throughput tissue grinder, followed by centrifugation at 10,000× *g* for 10 min. Then, 2 mL of the supernatant was aspirated in a glass test tube, and 2 mL of 0.5% thiobarbituric acid (TBA) was added to a boiling-water bath for 20 min, followed by cooling quickly, and spectrophotometers were used to measure the absorbance at 450, 532, and 600 nm. MDA content (μmol/gFW) = [6.452 × (OD532-OD600) − 0.559 × OD450] × VT/(V1 × FW), in which VT—the total volume of extracted enzyme solution, mL; V1—the total volume of extracted solution used for the determination, mL; FW—Fresh weight of the sample, g.

### 2.9. Statistical Analysis

In this study, a two-way analysis of variance (ANOVA) was conducted to evaluate the impact of combined drought and Cd stress on various performance and stress tolerance metrics of DSE strains, including biomass, Cd content, melanin, osmoregulatory substances, and antioxidant-enzyme activity. The statistical analysis was performed using SPSS 25.0 software, with significant differences identified through Duncan’s test (*p* < 0.05). Data processing was conducted using Excel 2021, while box scatter plots and correlation heat maps were generated with Origin Version 2021 software. The concentrations of PEG and Cd that corresponded to a 50% growth inhibition (IC_50_) for the DSE strains were determined utilizing GraphPad Prism version 8.0 software. Variance partitioning analysis (VPA) was implemented to dissect the effects of drought and Cd as individual factors on the performance and tolerance indices of DSE strains, employing the “vegan” package within RStudio version 1.3.1073 software. Furthermore, the influence of DSE strains on antioxidant, osmotic-regulatory, and drought-resistance indices was elucidated through a Structural Equation Model (SEM) using AMOS 21.0 software.

## 3. Results

### 3.1. Effect of Drought Stress on Morphology and Growth of DSE Colonies

**Figure 1:**
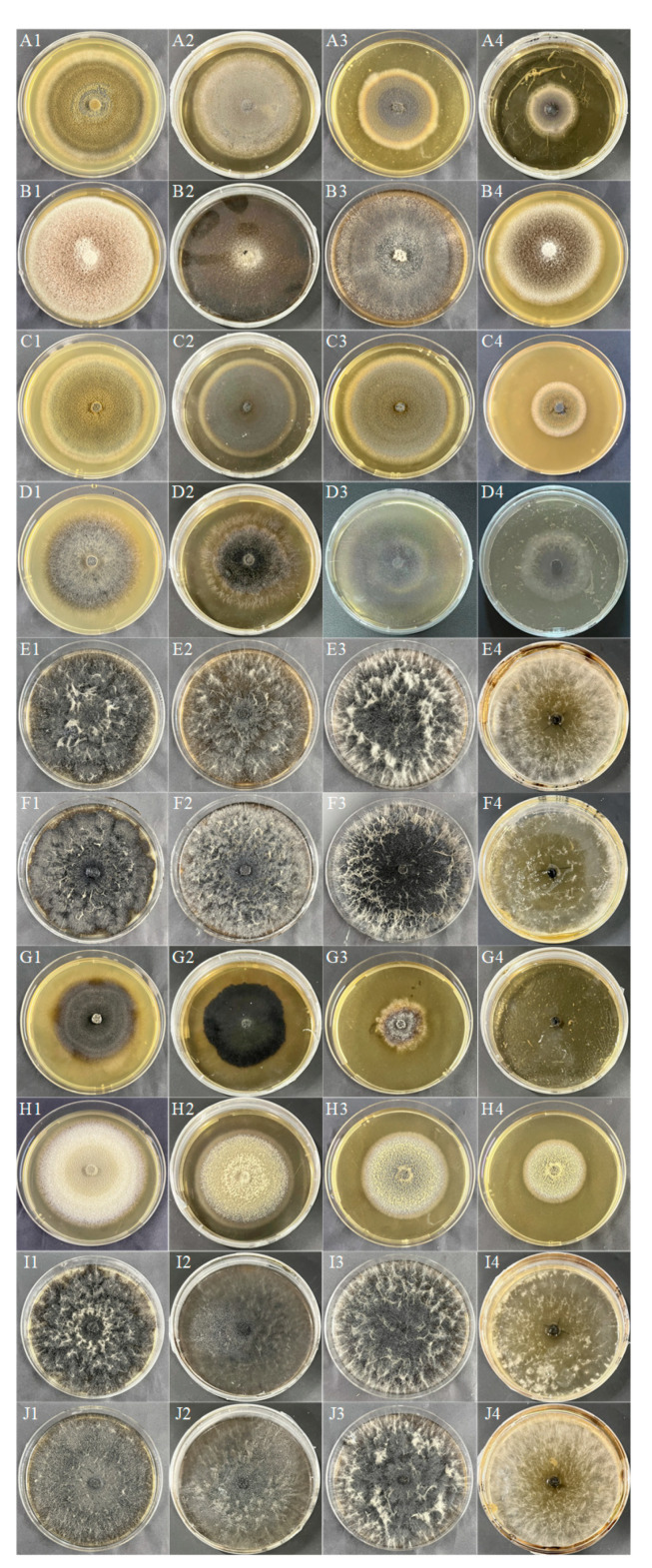
DSE colony morphology under different PEG concentrations. (**A**–**J**) stands for *M. cytisi* (*Mc*), *A. alstroemeriae* (*Aa*), *S. lupini (Sl*), *N. phragmitis* (*Np*), *P. chlamydocopiosa* (*Pc*), *M*. *phaseolina* (*Mp*’), *P. equi* (*Pe*), *A. tellustris* (*At*), *M. pseudophaseolina* (*Mp*), and *P. radicina* (*Pr*). **1**–**4** indicates PEG gradients of 0, 9, 18, and 27%, respectively.

**Figure 2:**
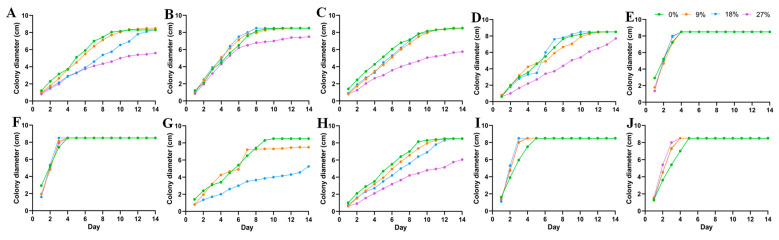
Colony growth diameters of 10 DSE strains under different PEG concentrations. (**A**–**J**) stands for *M. cytisi* (*Mc*), *A. alstroemeriae* (*Aa*), *S. lupini* (*Sl*), *N. phragmitis* (*Np*), *P. chlamydocopiosa* (*Pc*), *M. phaseolina* (*Mp’*), *P. equi* (*Pe*), *A. tellustris* (*At*), *M. pseudophaseolina* (*Mp*), and *P. radicina* (*Pr*).

**Figure 3:**
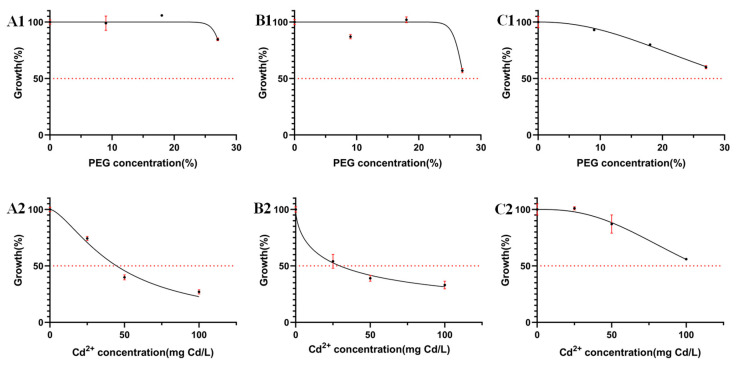
Effect of PEG concentration (**A1**–**C1**) and Cd concentration (**A2**–**C2**) on the mean and standard deviation (solid bars, *n* = 3) of the tolerance index (TI) of selected stress-tolerant strains measured in radial growth in PDA medium after 8 d of incubation. The solid line corresponds to the fitted logistic model used to determine the IC_50_ values. (**A**–**C**) stands for *A. alstroemeriae* (*Aa*), *N. phragmitis* (*Np*), and *A. tellustris* (*At*).

The colony morphology of 10 DSE strains under drought stress after 8 d of PDA medium cultivation is shown in Figure 1A–J. The colony growth rates of *At* decreased with the increase of drought gradient. *Aa*, *Np*, *Pc*, *Mp’*, and *Mp* increased and then decreased in colony growth rate with the increase of drought stress and were the first to reach the maximum diameter at a PEG concentration of 18% (Figure 2). *Pr* showed a faster growth rate under drought stress, and the mycelium cultured under 9%, 18%, and 27% PEG concentrations preferentially attained its maximum diameter on the fourth day. With the increase of PEG-6000 concentration, except *Pe*, all DSE strains showed different degrees of drought resistance. The tolerance index (TI) of DSE strains to varying PEG concentrations was assessed, along with the estimation of the 50% inhibitory concentration (IC_50_) after an 8-day incubation period. An incubation time of 8 d was used as an intermediate reference (Table 3). *Pc*, *Mp’*, *Mp*, and *Pr* strains had the highest tolerance to PEG concentrations. After 8 d of incubation in a PDA medium, *Aa* was the next most tolerant to PEG, with a TI of 0.85 at 27% PEG concentration. In the corresponding model, the IC50 estimate for *At* was 32.84% PEG concentration, which exceeds the highest concentration of PEG tested in the study (Figure 3(C1)).

### 3.2. Effect of Cd Stress on Morphology and Growth of DSE Colonies

**Figure 4:**
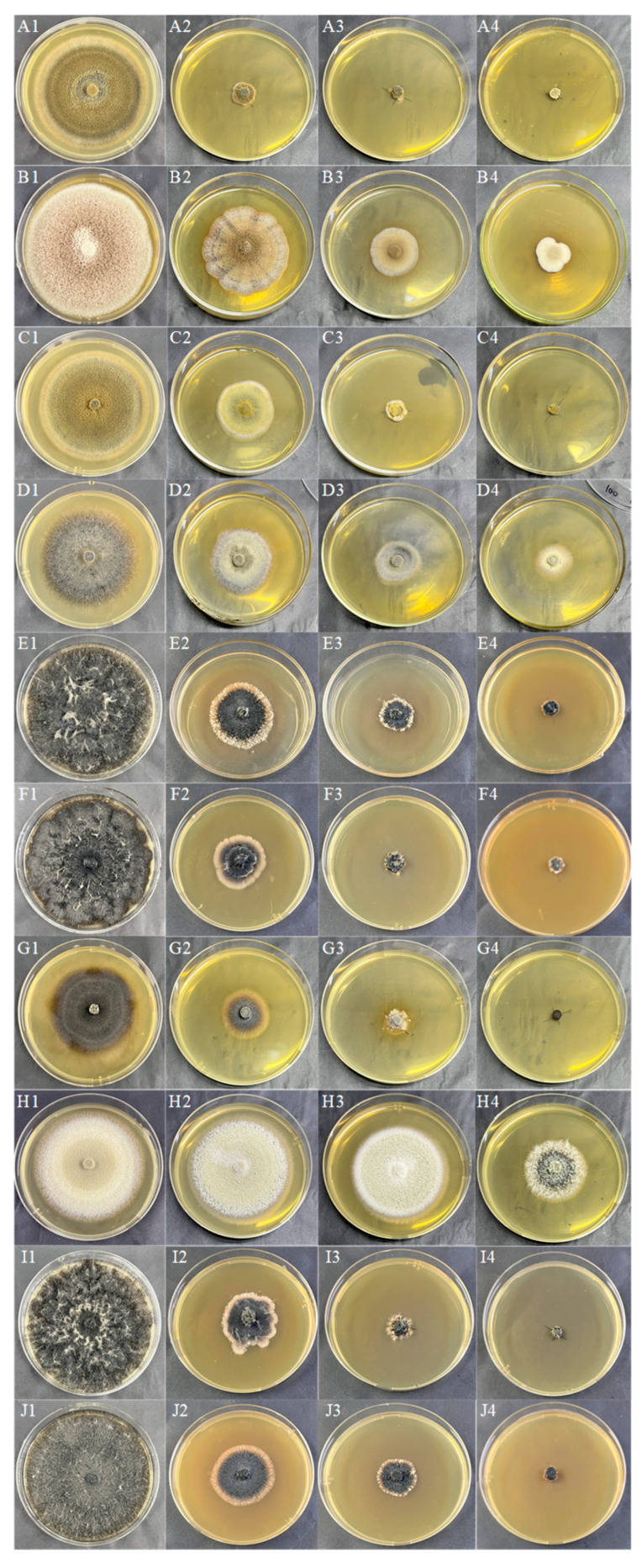
DSE colony morphology under different Cd stresses. (**A**–**J**) stands for *M. cytisi* (*Mc*), *A. alstroemeriae* (*Aa*), *S. lupini* (*Sl*), *N. phragmitis* (*Np*), *P. chlamydocopiosa* (*Pc*), *M. phaseolina* (*Mp’*), *P. equi* (*Pe*), *A. tellustris* (*At*), *M. pseudophaseolina* (*Mp*), and *P. radicina* (*Pr*). **1***–***4** indicates Cd stress gradients of 0, 25, 50, and 100 mg/L, respectively.

**Figure 5:**
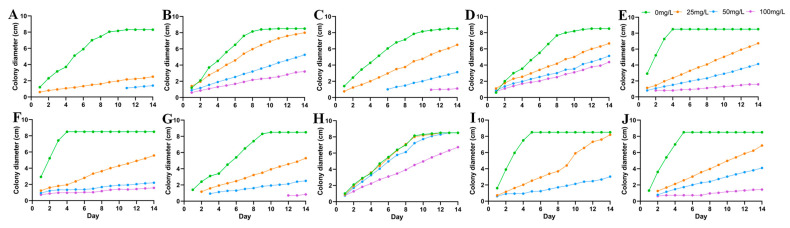
Growth diameters of 10 DSE strains under different Cd stresses. (**A**–**J**) stands for *M. cytisi* (*Mc*), *A. alstroemeriae* (*Aa*), *S. lupini* (*Sl*), *N. phragmitis* (*Np*), *P. chlamydocopiosa* (*Pc*), *M. phaseolina* (*Mp’*), *P. equi* (*Pe*), *A. tellustris* (*At*), *M. pseudophaseolina* (*Mp*), and *P. radicina* (*Pr*).

The colony morphology of 10 DSE strains under different Cd concentrations after 8d of PDA medium cultivation is presented in Figure 4A–J. With the increase of Cd stress, the adaptability changes of colony morphology varied among different strains, and the color of the *Mc*, *Aa*, *Sl*, *Np*, and *Pe* colonies gradually became lighter. The mycelia of *Pc*, *Mp’*, *At*, *Mp*, and *Pr* gradually changed from loose to dense and darkened in color. The colony diameter of the strain decreased with increasing Cd concentration. As the concentration of Cd rose, *Mc* and *Mp* stopped growing when the concentration reached 100 mg Cd/L. *Sl*, *Pc*, *Mp’, Pe*, and *Pr* grew slowly at 100 mg Cd/L. *Aa*, *Np*, and *At* remained growth-active at 100 mg Cd/L (Figure 5). The tolerance index (TI) of DSE strains to a range of Cd concentrations was determined, alongside the calculation of the 50% inhibitory concentration (IC_50_) following an 8-day period. An incubation time of 8 days was used as an intermediate reference (Table 3). The IC50 estimates for *Aa*, *Np*, and *At* were 48 mg Cd/L, 30 mg Cd/L, and 109 mg Cd/L, respectively (Figure 3(A2)–(C2)). From the one-factor Cd stress results, it was concluded that *Aa*, *Np*, and *At* had better Cd tolerance.

### 3.3. Effects of Combined Stress of Drought and Cd on Morphology and Physiological Indices of DSE Colonies

#### 3.3.1. Morphological Parameters and Biomass of DSE Colonies

**Figure 6:**
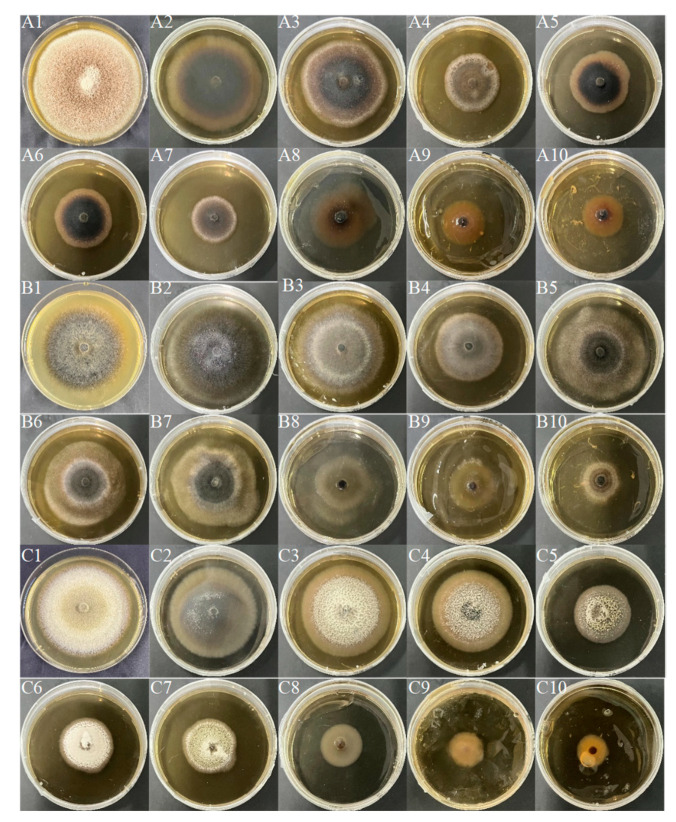
DSE colony morphology under different combined stress of drought and Cd. (**A**–**C**) stands for *A. alstroemeriae* (*Aa*), *N. phragmitis* (*Np*), and *A. tellustris* (*At*). **1**–**10** indicate that the combined stress gradients of drought and Cd are 0, 9% + 25 mg Cd/L, 9% + 50 mg Cd/L, 9% + 100 mg Cd/L, 18% + 25 mg Cd/L, 18% + 50 mg Cd/L, 18% + 100 mg Cd/L, 27% + 25 mg Cd/L, 27% + 50 mg Cd/L, and 27% + 100 mg Cd/L, respectively.

**Figure 7:**
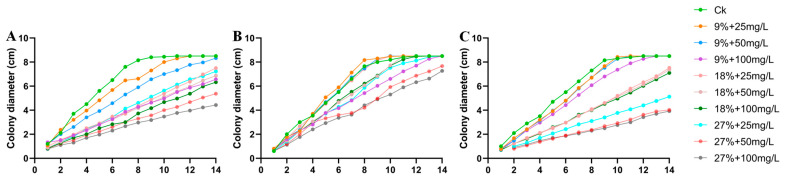
Colony diameter of three DSE strains under different combined stress of drought and Cd. (**A**–**C**) stands for *A. alstroemeriae* (*Aa*), *N. phragmitis* (*Np*), and *A. tellustris* (*At*). 1–10 indicate that the combined stress gradients of drought and Cd are 0, 9% + 25 mg Cd/L, 9% + 50 mg Cd/L, 9% + 100 mg Cd/L, 18% + 25 mg Cd/L, 18% + 50 mg Cd/L, 18% + 100 mg Cd/L, 27% + 25 mg Cd/L, 27% + 50 mg Cd/L, and 27% + 100 mg Cd/L, respectively.

Combined with the results of single-factor drought and Cd stress, three DSE strains *Aa*, *Np*, and *At*, were selected for a drought-and-Cd-combined stress test. After 8 d of PDA medium cultivation, the colony morphology of three DSE strains under different combined stresses of drought and Cd was measured (Figure 6). The colony color of *Aa* was grayish-white and dense when there was no stress. The colony color changed from black to grayish-white with the increase of stress degree (Figure 6A). The *Np* strain had a black colony color with aerial mycelium on the surface in the absence of stress, and with increasing stress, the mycelium on the edge of the colony was sparse, the edge was covered with white downy hairs, and the aerial mycelium increased. *Np* grew fastest after 8 days at a 9% + 25 mg Cd/L concentration (Figure 6B). In the absence of stress, the color of the colonies of the *At* strain was whitish with dense colonies. At a concentration of 9% + 25 mg Cd/L, colonies darkened in color, and with increasing stress, the color of the colonies changed from black to white, with white tomentum in the middle (Figure 6C). The colony diameter of the three DSE strains is shown in Figure 7. As combined stress increased, the colony diameter of *Aa* declined in comparison to the control; *Np* preferentially culminated in a maximum diameter after 10 d at 9% + 50 mg Cd/L and 18% + 25 mg Cd/L combined stress. The colony diameter of *At* strains revealed a tendency of first growing and then declining with the increase of combined stress, and the diameter preferentially culminated in a maximum at 9% + 25 mg Cd/L combined stress. The results showed that the *Np* strain had strong drought-and-Cd-combined stress tolerance.

**Figure 8:**
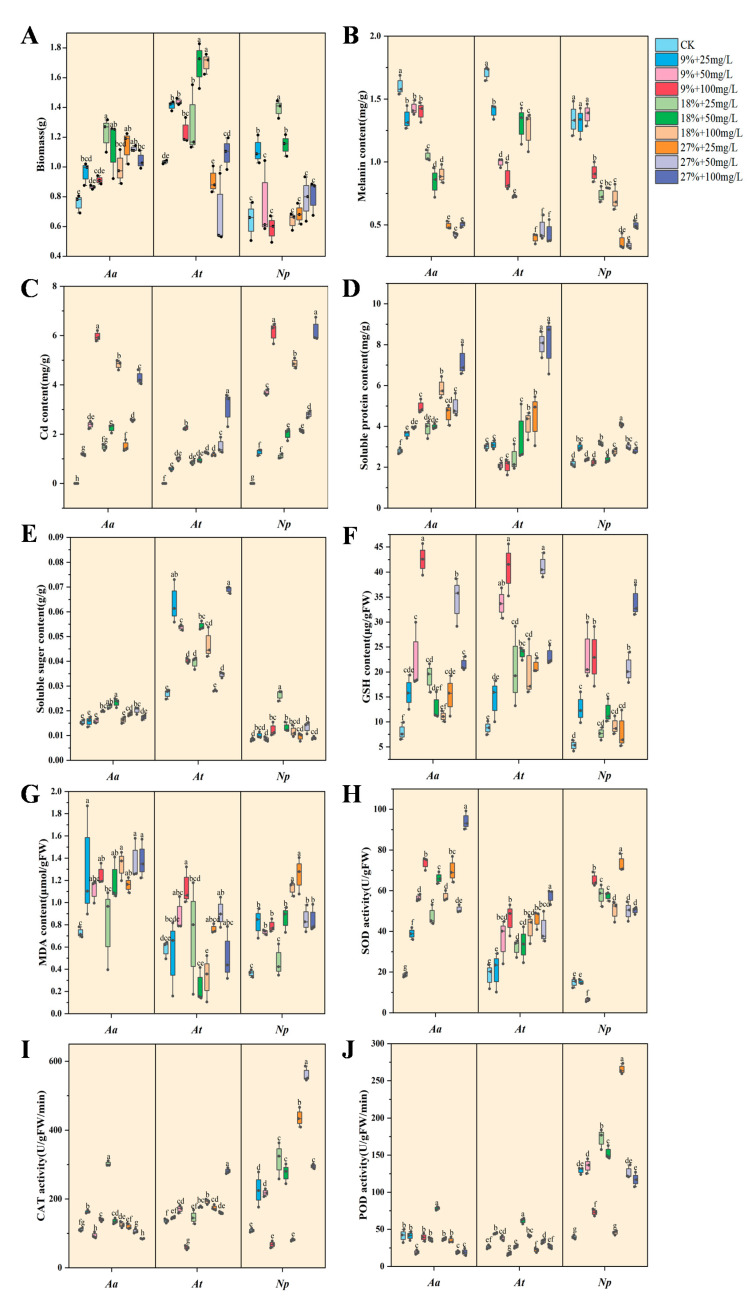
Physiological and growth indexes of DSE strains in liquid cultures. The abbreviations in the figure are *A. alstroemeriae* (*Aa*), *A. tellustris* (*At*), and *N. phragmitis* (*Np*). Biomass (**A**), melanin content (**B**), Cd content (**C**), soluble protein content (**D**), soluble sugar content (**E**), GSH content (**F**), MDA content (**G**), SOD activity (**H**), CAT activity (**I**), and POD activity (**J**) of DSE under drought and Cd stress. Means followed by the different letter(s) within each column are significantly different at *p* < 0.05.

The biomass of *Aa* was higher under all combined stress than under the control treatment, but with the aggravation of combined stress, the biomass of *Aa* increased first and then decreased and reached the highest value of 1.23 g at the combined concentration of 18% + 25 mg Cd/L, a significant increase of 61.84% compared with the control (Figure 8A). The biomass of *At* and *Np* revealed a growing and subsequently declining trend with the aggravation of combined stress. The *At* reached the highest biomass of 1.71 g at the combined concentration of 18% + 50 mg Cd/L, which was a significant increase of 64.42% compared with the control. The *Np* reached the highest biomass of 1.39 g at the combined concentration of 18% + 25 mg Cd/L, which was a significant increase of 117.19% compared with the control. The biomass of *Np* increased at 9% + 25 mg Cd/L, 18% + 25 mg Cd/L, and 18% + 50 mg Cd/L compared with the control, but there was no significant difference between other treatments and the control.

#### 3.3.2. Melanin and Cd Content of DSE Strains

With a rise in stress level, the melanin content of *Np* first increased before declining and attained the maximum value of 1.38 mg/g at the combined concentration of 9% + 50 mg Cd/L, which increased by 2.84% as compared to the control (Figure 8B). However, the melanin content of *Aa* and *At* decreased with the enhancement of combined stress, and the difference was significant compared with the control. With an increase in Cd concentration, the Cd content of DSE strains gradually increased (Figure 8C). The Cd content of *Aa* was 1.18, 2.38, 5.97, 1.47, 2.25, 4.82, 1.51, 2.57, and 4.28 mg/g, the Cd content of *At* was 0.59, 1.02, 2.20, 0.88, 0.94, 1.27, 1.23, 1.78, and 2.97 mg/g, and the Cd content of *Np* was 1.28, 3.69, 6.15, 1.09, 2.01, 4.87, 2.14, 2.81, and 6.18 mg/g, respectively.

#### 3.3.3. Soluble Substance Content of DSE Strains

The soluble protein content of *Aa* and *Np* strains increased with the increase of combined stress (Figure 8D). Compared with the control, the soluble protein content of *Aa* under all combined stress treatments increased and reached the maximum value in 27% + 100 mg Cd/L treatment, which was 2.55 times that of the control and increased by 155.36% compared with the control. At the combined concentration of 18% + 100 mg Cd/L, 27% + 25 mg Cd/L, 27% + 50 mg Cd/L, and 27% + 100 mg Cd/L, the soluble protein content of *At* was increased compared to the control and reached the maximum value at the combined concentration of 27% + 100 mg Cd/L, which was increased by 181.94% in comparison to the control. No significant differences were observed between the other treatments and the control group. However, the soluble protein content in the *Np* peaked under the 27% + 25 mg/L Cd treatment, exhibiting a substantial increase of 87.16% relative to the control.

The soluble sugar content of *Aa* and *Np* strains showed an increasing and then decreasing trend with the increase of combined stress (Figure 8E). The combined concentration of *Aa* was maximum at 18% + 50 mg Cd/L, reaching a maximum of 0.023 g/g. The *Np* was higher than the control at the combined concentration of 18% + 25 mg Cd/L, reaching a maximum of 0.026 g/g. The soluble sugar content of *At* was higher in the other treatments (except 27% + 25 mg Cd/L) when compared with the control. The maximum value was 0.07 g/g when the combined concentration was 27% + 100 mg Cd/L, which was 2.33 times that of the control.

#### 3.3.4. GSH and MDA Content of DSE Strains

The GSH content of DSE strains was increased under all the combined stress treatments compared to the control (Figure 8F). The *Aa* reached the maximum value of 42.56 μg/g FW at the combined concentration of 9% + 100 mg Cd/L. At a combined concentration of 27% + 50 mg Cd/L, the *At* reached a maximum value of 37.88 μg/g. The combined concentration of *Np* was 27% + 100 mg Cd/L, which was 6.41 times higher than the control.

The MDA content of *Aa* and *Np* increased with each treatment compared with the control except for the combined concentration of 18% + 25 mg Cd/L (Figure 8G). The MDA content of *Aa* reached the maximum value in the 27% + 100 mg Cd/L treatment, which was 1.89 times that of the control. The MDA content of the *Np* reached a maximum of 1.25 μmol/g FW in the 27% + 25 mg Cd/L treatment, which was 3.47 times higher than the control. The MDA content of *At* reached a maximum value of 1.09 μmol/g FW at 9% + 100 mg Cd/L combined concentration, which increased by 94.64% compared with the control.

#### 3.3.5. Antioxidant Enzyme Activity of DSE Strains

With the intensification of combined stress, the SOD activity of *Aa* and *At* increased compared with the control (Figure 8H). At a combined concentration of 27% + 100 mg Cd/L, the SOD activity of *Aa* and *At* was more than that of the control, and the SOD activity showed the maximum value, which increased by 407.22% and 241.12%, respectively. The SOD activity of *Np* showed a decreasing and then increasing trend with the increase in the degree of stress and reached the maximum value at 27% + 25 mg Cd/L, which increased 390.87% compared with the control. Compared with the control, SOD activity decreased by 56.78% when the combined concentration was 9% + 50 mg Cd/L, and its activity was greatly reduced.

The CAT activity of *Aa* showed an increasing and then decreasing trend with the increase of combined stress and reached the maximum value at 18% + 25 mg Cd/L concentration, which increased by 174.25% compared with the control (Figure 8I). In addition to 9% + 100 mg Cd/L treatment, CAT activity of *At* increased compared with the control and reached the maximum value at 27% + 100 mg Cd/L combined concentration, which was 2.07 times that of the control. Except for the combined concentrations of 9% + 100 mg Cd/L and 18% + 100 mg Cd/L, the *Np* showed an increase in CAT activity in other treatments compared to the control and reached a maximum of 560.55 U/g FW/min at 27% + 50 mg Cd/L, which was an increase of 416.97% compared to the control.

The POD activity of *Aa* reached the maximum value of 78.45 U/g FW/min under moderate stress (18% + 50 mg Cd/L), which increased by 89.04% compared with the control (Figure 8J). Under 50 mg Cd/L stress, POD activity of *At* under 9%, 18%, and 27% drought stress was increased compared with the control, which were 46.19%, 134.16%, and 29.17% of the control, respectively. The POD activity of *Np* was higher than that of the control under all combined stress treatments. POD activity reached the maximum value of 265.41 U/g FW/min under the 27% + 25 mg Cd/L treatment, which was increased by 573.12% compared with the control. The POD activity of *Np* under 25 mg Cd/L stress and 9%, 18%, and 27% drought stress was increased by 227.97%, 338.22%, and 573.12%, respectively, compared with the control.

### 3.4. Relationship between DSE Growth and Physiological Indicators

**Figure 9:**
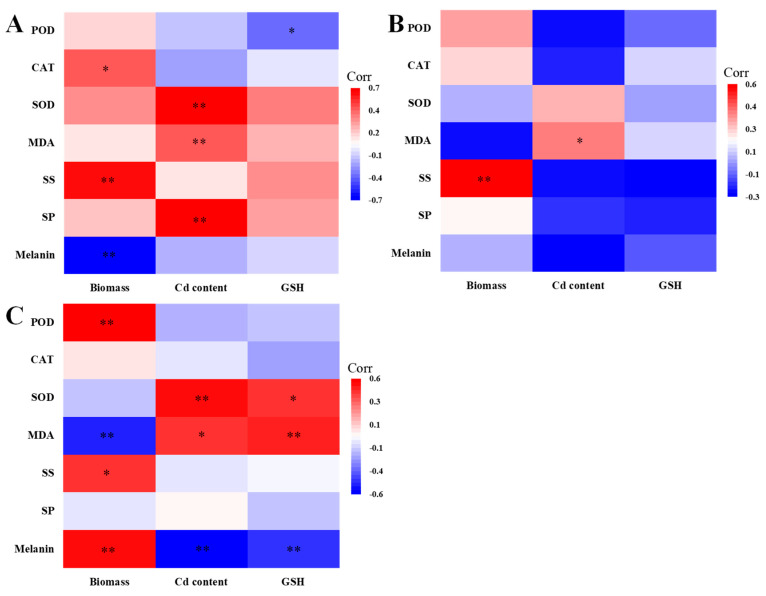
Relationship between DSE growth and physiological indicators. (**A**–**C**) stands for *A. alstroemeriae* (*Aa*), *N. phragmitis* (*Np*), and *A. tellustris* (*At*). SS represents soluble sugar, and SP represents soluble protein. The various symbols above the bars indicate significant differences between DSE growth and physiological indices (* *p* < 0.05, ** *p* < 0.01).

The relationship between biomass, Cd content, GSH and melanin, SP, SS, MDA, SOD, CAT, and POD was further analyzed for each DSE (Figure 9), and the correlations between the individual metrics varied depending on the DSE.

The biomass of *Aa* was positively correlated with CAT activity and soluble sugar content and negatively correlated with melanin content. The Cd content of *Aa* was positively correlated with SOD activity, MDA, and soluble protein content. GSH content of *Aa* was negatively correlated with POD activity (Figure 9A). The biomass of *Np* was positively correlated with the soluble sugar content. The Cd content of *Np* was positively correlated with MDA content (Figure 9B). The biomass of *At* was positively correlated with POD activity, soluble sugar, and melanin content but negatively correlated with MDA content. Cd and GSH content of *At* were positively correlated with SOD activity and MDA content and negatively correlated with melanin content (Figure 9C).

### 3.5. Variation Partitioning Analyses

**Figure 10:**
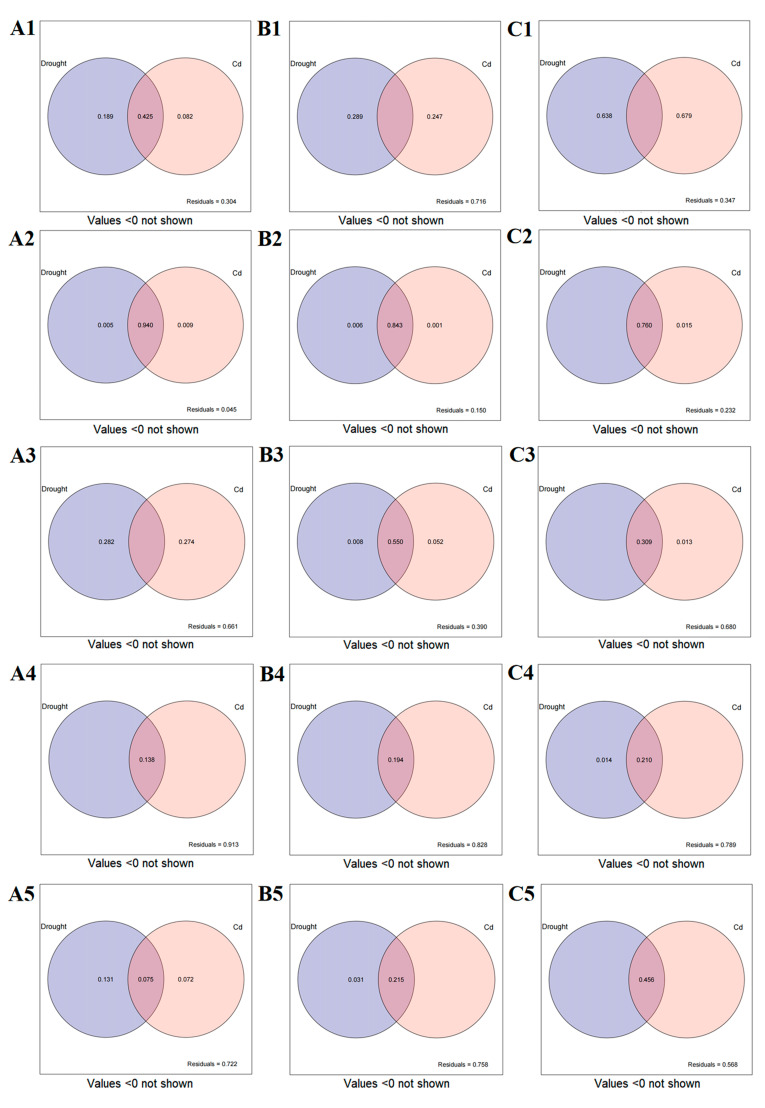
The variance-partitioning analysis of drought and Cd stress on growth and physiological indices of DSE. (**A**–**C**) represents the variance-partitioning analysis plots of *A. alstroemeriae* (*Aa*), *N. phragmitis* (*Np*), and *A. tellustris* (*At*) under combined stress, respectively. **1** represents the effect of drought and Cd stress on the biomass. **2** represents the effect of drought and Cd stress on the melanin content. **3** represents the effect of drought and Cd combined on the antioxidant-enzyme activity. **4** represents the effect of drought and Cd stress on the SP, SS, MDA, and GSH. **5** represents the effect of drought and Cd stress on the Cd content.

The effects of drought-and-Cd stress on the physiological and growth indicators of DSE were estimated by variance partitioning analysis, and the contribution rates of various factors to the differences of DSE strains were quantitatively assessed (Figure 10). The combined explanation of drought-and-Cd stress on the biomass of *Aa* was 69.6%. The individual explanations were 18.9% and 8.2%, respectively. The interaction between drought and Cd accounted for 42.5% (Figure 10(A1)). The combined explanation of drought-and-Cd stress on the biomass of *Np* was 28.4%, and the individual explanations were 28.9% and 24.7%, respectively (Figure 10(B1)). The combined explanation of drought-and-Cd stress on the biomass of *At* was 65.3%. The individual explanations were 63.8% and 67.9%, respectively (Figure 10(C1)).

The variance in melanin production by strain *Aa*, attributed to combined drought-and-Cd stress, was explained by 95.5% of the model, with individual contributions of 0.5% for drought and 0.9% for Cd stress alone and an interaction effect accounting for 94% (Figure 10(A2)). For strain *Np*, the combined stress factors explained 85% of the variance in melanin levels, with individual contributions of 0.6% for drought and 0.1% for Cd stress, and the interaction effect was responsible for 84.3% (Figure 10(B2)). In the case of strain *At*, the combined stress factors explained 76.8% of the variance in melanin production, with Cd stress alone accounting for 1.5% and the interaction between drought and Cd stress contributing to 76% of the explained variance (Figure 10(C2)).

The impact of combined drought-and-Cd stress on the antioxidant-enzyme activity in the *Aa* strain was found to be 33.9%, with individual contributions of 28.2% attributed to drought and 27.4% to Cd stress alone (Figure 10(A3)). For the *Np* strain, the combined stress factors explained 61% of the variance in antioxidant-enzyme activity, with individual contributions of 0.8% for drought and 5.2% for Cd stress, and the interaction between the two stressors accounted for 55% (Figure 10(B3)). In the case of the *At* strain, the combined stress factors explained 32% of the variance in antioxidant-enzyme activities, with Cd stress alone contributing 1.3% and the interaction between drought and Cd stress accounting for 30.9% (Figure 10(C3)).

The effects of combined drought and Cd stress on the physiological indices of strains *Aa*, *Np*, and *At* were 8.7%, 17.2%, and 21.1%, respectively. The interactions between the two stressors were 13.8%, 19.4%, and 21%, respectively, with Cd stress alone explaining 1.4% of the physiological indices in the *At* strain (Figure 10(A4)–(C4)).

In terms of Cd content, the combined stress factors explained 27.8% of the variance in the *Aa* strain, with individual contributions of 13.1% for drought and 7.2% for Cd stress, and the interaction between the two factors was 7.5% (Figure 10(A5)). For the *Np* strain, the combined stress factors explained 24.2% of the variance in Cd content, with drought stress contributing 3.1% and the interaction between drought and Cd stress accounting for 21.5% (Figure 10(B5)). For the *At* strain, the combined explanation of drought and Cd stress on Cd content was 43.2%, with the interaction between the two stressors contributing a significant 45.6% (Figure 10(C5)).

### 3.6. Correlation Analyses

**Figure 11:**
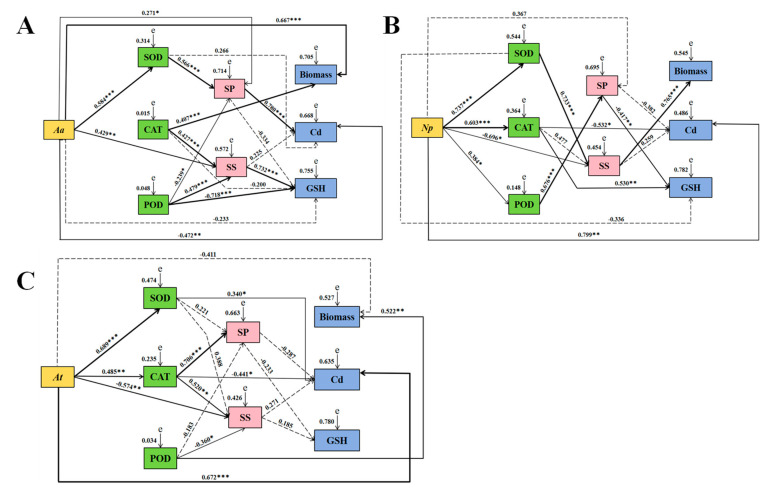
Structural-equation modeling of causal relationships between DSE and enzyme activity, soluble matter, biomass, Cd, and GSH content. (**A**–**C**) represents structural-equation models of *A. alstroemeriae* (*Aa*), *N. phragmitis* (*Np*), and *A. tellustris* (*At*), respectively. SS represents soluble sugar, and SP represents soluble protein. Results of fitness of influencing factors of *Aa*: (**A**) *χ^2^* = 8.500, *df* = 6, *p* = 0.004, RMSEA (root mean square error of approximation) = 0.120, GFI (goodness-of-fit index) = 0.942, IFI (incremental fit index) = 0.987, CFI (comparative fit index) = 0.984; Results of fitness of influencing factors of *Np*: (**B**) *χ^2^* = 28.978, *df* = 6, *p* = 0.001, RMSEA = 0.363, GFI = 0.870, IFI = 0.883, CFI = 0.862; Results of fitness of influencing factors of *At*: (**C**) *χ*^2^ = 21.358, *df* = 6, *p* = 0.002, RMSEA = 0.297, GFI = 0.877, IFI = 0.913, CFI = 0.895. Whether the path of action between various factors was significant or not is indicated by the solid and dotted lines, respectively. The numbers near the arrows represent the normalized path coefficients, and the width of the solid line represents the strength of the effect between the various factors (* *p* < 0.05, ** *p* < 0.01, and *** *p* < 0.001).

Based on the correlation according to the coefficients of correlation (R-values), the association between DSE and all examined parameters was evaluated using a SEM model. *Aa* positively influenced SOD, soluble sugar, and biomass and negatively affected Cd content. The SOD activity of *Aa* had a positive impact on soluble protein. The CAT activity of *Aa* had a positive impact on soluble sugar and biomass. The POD activity of *Aa* had positive effects on soluble sugar and adverse impacts on soluble protein and GSH. The soluble protein of *Aa* had a positive effect on Cd content (Figure 11A).

The *Np* had a positive effect on SOD, CAT, POD, and Cd content. *Np* negatively affected soluble sugars. The SOD of *Np* positively influenced soluble sugars. The CAT of *Np* negatively affected Cd content. The POD of *Np* positively influenced soluble protein. The soluble protein of *Np* had a negative effect on GSH. The soluble sugars of *Np* positively influenced biomass (Figure 11B).

The *At* positively influenced SOD, CAT, and Cd content. *At* negatively affected soluble sugars. The SOD activity of *At* positively influenced Cd content. The CAT activity of *At* positively influenced soluble protein and soluble sugar and negatively affected Cd content. The POD activity of *At* positively influenced biomass and negatively affected soluble sugar (Figure 11C).

### 3.7. Growth Parameter

**Figure 12:**
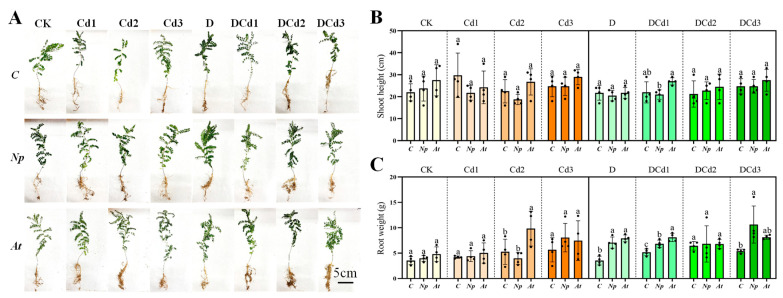
Effect of different DSEs on growth-morphological parameters of *Astragalus membranaceus* seedlings’ growth under synergistic stress of drought and Cd. Growth picture (**A**), shoot height (**B**), and root weight (**C**) of *A. membranaceus* under drought and Cd stress. The abbreviations in the figure are non-inoculated plants(*C*), *N. phragmitis* (*Np*), and *A. tellustris* (*At*). Means followed by the different letter(s) within each column are significantly different at *p* < 0.05.

DSE had significant effects on the growth of *A. membranaceus* seedlings under drought and Cd stress (Figure 12A). Specifically, the effect of *Np* and *At* inoculation on shoot height was not significant (Figure 12B). Under Cd2, D, and DCd1 treatments, inoculation with *At* significantly increased root weight by 86.88, 124.36, and 56.78%, respectively, compared with non-inoculated counterparts. Under D, DCd1 and DCd3 treatments, inoculation with *Np* significantly increased root weight by 100.57, 32.54 and 97.04%, respectively, compared with non-inoculated counterparts (Figure 12C).

## 4. Discussion

### 4.1. Effects of Drought Stress on the Performance of DSE

All nine strains of DSE, except *Pe*, grew well under a drought-stress environment in this study. The growth rates of *Aa*, *Np*, *Pc*, *Mp’*, and *Mp* surpassed those of the control under an 18% drought stress. Corresponding research has indicated that a PEG-6000 concentration ranging from 10% to 20% is conducive to the biomass accumulation of ectomycorrhizal fungi [43]. Under mild or moderate drought conditions, most DSE strains exhibited darkened colonies, whereas under severe drought stress, most DSE strains exhibited lightened colonies. This phenotypic plasticity is likely an adaptive response of DSE strains to stressful environments, potentially enhancing the efficiency of nutrient and water uptake [44]. Resistant cultivars can drive divergence in the ecological roles of the cultivated fungi *Mortierella alpina* and *Epicoccum nigrum*, and successful colonization of root surfaces by *M. alpina* enhances the resilience of wheat drought stress through the activation of drought-responsive genes [45]. The combination of the arbuscular mycorrhizal fungi (AMF) (*Glomus* spp.) with energy grasses also improves the adaptation of *Saccharum arundinaceum* to marginal lands with drought-affected soils [46]. It has been shown that the symbiosis of AMF enhanced the tobacco plants’ secondary metabolism and overall growth pattern under severe drought stress [47]. It has been shown that two Cd-tolerant and plant-growth-promoting actinomycete strains, *Streptomyces* sp. and *Nocardiopsis* sp., were isolated from metal-contaminated soils. Both actinomycete strains can be used as effective agents for phytoremediation of soil contaminated with Cd under drought conditions [48].

### 4.2. Effects of Cd Stress on the Performance of DSE

With the increasing concentration of Cd, the alterations in colony morphology among strains exhibited strain-specific responses. Notably, strains *Aa*, *Np*, and *At* demonstrated sustained activity even at 100 mg Cd/L, indicative of their superior Cd tolerance. This resilience is likely attributed to the inherent capacity of certain DSEs to strongly adsorb heavy metals, thereby impeding the mobility of heavy-metal ions. Among these, *At* displayed the most rapid growth rate under varying Cd stress levels. The observed differences in tolerance and growth kinetics are postulated to stem from the distinct biochemical attributes and physiological adaptations of DSEs in response to Cd-induced stress [49]. The IC50 estimates for *Aa*, *Np*, and *At* were 48 mg Cd/L, 30 mg Cd/L, and 109 mg Cd/L, respectively. Studies have reported that endophytic fungi isolated from barley roots, such as *Alternaria* sp., exhibit exceptional tolerance to Cd, a finding that corroborates the results presented here [50]. The study showed that *Np* was isolated from saline areas [51]. In this study, *Neocamarosporium* sp. was also screened for Cd tolerance, which enriched the strain resource library. Medina-Armijo et al. [31] screened *Exophiala crusticola* for Cr tolerance. DSE species were isolated and characterized from the roots of *Medicago sativa* and *Ammopiptanthus mongolicus*, and the results showed enhanced growth and tolerance to Cd in the host plants [52]. Furthermore, by promoting the conversion of Cd into chemical forms that have low activity, DSE inoculation has been demonstrated to increase plants’ ability to withstand Cd stress and may mitigate the detrimental effects of Cd toxicity on plant growth [19]. Lin et al. [53] characterized the bioaccumulation of Zn and Cd by *Streptomyces zinciresistens*. Xue and Wang [54] found that inoculating soil with Cd-resistant *Actinomycetes* flora can reduce Cd accumulation in rice plants.

### 4.3. Effects of Combined Drought and Cd Stress on the Performance of DSE

In the current study, solid-plate-culture experiments showed that *Np* and *At* promoted fungal growth under low combined stress with increasing combined concentration, while high combined stress showed inhibition for the growth of DSE strains. The reason may be that DSEs are adaptive to the combined stress environment, which showed low promotion and high inhibition, and *Np* had more drought-and-Cd-combined tolerance. Liquid-shaker-culture experiments showed that the biomass of *Aa*, *At*, and *Np* strains achieved maximum values at 18% + 25 mg Cd/L, 18% + 50 mg Cd/L, and 18% + 25 mg Cd/L, respectively. Therefore, the moderately stressed might be more suitable for the growth of DSE strains [55].

Melanin, a crucial constituent of the cell wall in DSEs, serves to decelerate the rate of cellular water loss, thereby augmenting the fungus’s survival and competitive edge in harsh environmental conditions [56,57,58]. However, in this study, with the increase of combined stress, the melanin content of *Aa*, *At*, and *Np* strains decreased, which indicated that the melanin content of different DSE strains had different effects on improving the stress resistance of fungi. The results showed that melanin accumulation was not an important character of heavy-metal tolerance in DSEs [59].

Glutathione (GSH) is an important antioxidant in living organisms, which not only scavenges free radicals and suboxide ion and reduces oxidative stress but also facilitates the maintenance of cellular homeostasis and contributes to the detoxification of harmful substances such as Cd [60,61]. In this study, the GSH content in strains *Aa*, *At*, and *Np* was observed to increase under all combined stress treatments when compared to the control group. However, as the intensity of the combined stress escalated, the GSH content in strains *Aa* and *At* initially rose and then declined. This biphasic response may be attributed to the role of GSH in cellular defense mechanisms, where it participates in the neutralization of reactive oxygen species (ROS). The combined stress likely elevated intracellular GSH levels, which, given its sulfhydryl structure, can react with ROS. Consequently, as GSH becomes engaged in these detoxification processes, its cellular concentration diminishes. A related study showed that two plants of the *Atriplex atacamensis* and *A. halimus* further reduced growth parameters but not GSH and proline contents under the combined stress of Cu, NaCl, and PEG, showing positive tolerance responses [62]. Furthermore, DSE has been shown to enhance antioxidant activity in plants, leading to increased levels of GSH and putrescine under heat-stress conditions [63]. Overall, this research indicates that DSEs play a significant role in enhancing plant tolerance to environmental stresses through mechanisms involving GSH and other antioxidants.

MDA, which membrane lipids create in reaction to reactive-oxygen species, is a trustworthy indicator of the degree of damage to the plasma membrane [64,65]. In this study, the MDA content of *Aa* and *Np* increased under all combined stress. The MDA content of *At* decreased compared with the control under moderate combined stress, indicating that *At* had a certain ability to weaken the accumulation of membrane-lipid-peroxidation products to resist the adverse effects of combined stress, and DSE cells were damaged when it exceeded a certain range. As shown by the correlation heat map, there was a positive connection between the MDA content of *Aa*, *Np*, *At* and the Cd concentration. It has been shown that there is a positive correlation between leaf MDA content and Cd concentration in the substrate [13], which is similar to the results of this study.

Organisms can adapt to a stressful environment by adding the content of soluble proteins and soluble sugars, thus raising the concentration of cell fluid [66,67,68]. The study findings demonstrate that the soluble protein content of DSE strains varied with increasing levels of combined stress. The soluble protein content in strains *At* and *Np* exhibited a pattern of decrease followed by an increase under the combined stress conditions, in contrast to the control group. The soluble protein content in strain *Aa* consistently increased across all stress treatments. The soluble sugar content of DSE strains was higher than that of the control treatment under all combined stress. Both *Aa* and *Np* reached their maximum values under moderate compound stress, whereas the soluble sugar content of *At* reached its maximum value under heavy combined stress. Organisms often adapt to stress-induced osmotic imbalances by actively augmenting the levels of osmoregulatory substances, which serves to ameliorate the detrimental impacts of heavy-metal exposure.

CAT, SOD, and POD are the most important antioxidant enzymes that can remove ROS free radicals in bacteria to protect cell membranes from various stress damage [69,70]. It has been proven that under extreme stress, DSE isolated from the root system of desert plants increased SOD activity [71]. In the current study, under 27% + 100 mg Cd/L treatment, the SOD activity of the *Aa* and *At* strains was greatly increased, suggesting that the strains can stimulate SOD activity to reduce toxic accumulation under combined stress. In contrast to the control, the SOD activity of *Np* demonstrated a pattern of decreasing and then increasing with the strengthening of the combined stress, and the CAT and POD of *Np* showed higher activities compared to both *Aa* and *At*. The *Aa* and *At* showed lower CAT and POD activities compared to the control at a combined concentration of 27% + 100 mg Cd/L and 9% + 100 mg Cd/L, respectively. Possibly due to the accumulation of H_2_O_2_ from DSE in the organisms caused by the combined stress, the oxidative system in the organisms was exacerbated. The CAT activity of the plants inoculated with DSE increased at all Cd concentrations, implying that this enzyme plays a crucial role in DSE-induced protection against oxidative stress in plants [13]. Tyagi et al. [72] discovered that when wheat plants were under drought stress, AM fungi increased the SOD activity. Additionally, the study by Muhammad et al. [73,74] indicated that melatonin significantly increased the activity of POD, CAT, and SOD in maize seedlings under drought stress, leading to improved drought tolerance. Overall, these studies emphasize the significance of antioxidant enzymes, including POD, CAT, and SOD, in increasing the resistance of plants to drought and other environmental stresses. The SEM model analysis revealed that *Np* and *At* could positively affect SOD and CAT activities and synergistically resist the combined stress.

### 4.4. Effects of DSE on Growth Performance of Astragalus membranaceus under Drought and Cd Stress

DSE is widespread in natural ecosystems and can help plants to resist a variety of stressful environments, especially under harsh conditions (e.g.**,** saline, contaminated habitats, and arid ecosystems) [9]. Inoculation with DSE is beneficial for plant growth and resistance, but different DSE strains vary in their ability to promote host plants [75]. It was shown that under moderate- and high-salt stress conditions, *P. macrospinosa* and *Cadophora* sp. improved the shoot and root growth of tomato plants after 6 weeks of inoculation [76]. Under single and synergistic stresses of drought and salt, inoculation with *F. mosseae* significantly increased the fresh weight of hemp plants. However, inoculation with *F. Mosseae* had no significant effect on hemp plant height [77]. The study showed that inoculation with *N. phragmitis* under drought stress significantly increased the plant height of *Lycium ruthenicum* [78]. Compared with non-inoculated treatments, there was no significant difference in inoculation with *N. phragmitis* on the shoot height of *A. membranaceus* in all treatments of this experiment. It may be that the same DSE shows different effects on different plants. However, inoculation with *N. phragmitis* under D, DCd1, and DCd3 treatments significantly increased the root weight of *A. membranaceus*. Compared with non-inoculated treatments, root weight was significantly increased inoculation of *A. tellustris* under Cd2, D, and DCd1 treatments. The effect of inoculation with DSE on the growth of *A. membranaceus* under drought and Cd stress varied depending on the type of DSE but was generally beneficial.

## 5. Conclusions

In this study, we found that the colony growth rates of *A. alstroemeriae*, *N. phragmitis*, *P. chlamydocopiosa*, *M. phaseolina*, and *M. pseudophaseolina* were the first to reach the maximum diameter at a PEG concentration of 18%. *A. alstroemeriae*, *N. phragmitis*, and *A. tellustris* still possessed growth activity as the concentration of Cd rose. In addition, we investigated the performance and tolerance parameters of *A. alstroemeriae*, *N. phragmitis*, and *A. tellustris* strains under combined drought and Cd stress. The results of solid cultures under combined stress showed that the growth rate of *N. phragmitis* was significantly better than that of other strains. In the liquid culture condition, *A. alstroemeriae* positively influenced biomass and negatively affected Cd content, while *N. phragmitis* and *A. tellustris* positively influenced Cd content. *A. alstroemeriae*, *N. phragmitis*, and *A. tellustris* positively influenced SOD activity, and MDA content was positively correlated with Cd content. The GSH contents of *A. alstroemeriae*, *N. phragmitis*, and *A. tellustris* were increased under the combined stress of drought and Cd. Inoculation of *N. phragmitis* and *A. tellustris* promotes plant growth under synergistic drought and Cd stress. The utilization of DSEs to enhance plant growth under different combined stresses of drought and Cd has significant potential in the future.

## 6. Patents

A patent entitled “A DSE strain with drought-and-Cd-combined tolerance and its application in improving plant stress resistance” has been published and is currently in the application stage.

## Figures and Tables

**Table 1:** List of DSE fungal strains used in this study.

Strain	Host Plant	Geographic Location	Acc. nr
*Microsphaeropsis cytisi*	*Glycyrrhiza uralensis*	Chifeng in Inner Mongolia	ON413886
		Autonomous Region	
*Stagonosporopsis lupini*	*Glycyrrhiza uralensis*	Chifeng in Inner Mongolia	OP363602
		Autonomous Region	
*Neocamarosporium phragmitis*	*Glycyrrhiza uralensis*	Jingtai, Gansu Province	ON413888
*Alternaria alstroemeriae*	*Isatis indigotica*	Anguo, Hebei Province	MZ505449
*Macrophomina pseudophaseolina*	*Astragalus membranaceus*	Anguo, Hebei Province	MZ506881
*Paraphoma radicina*	*Astragalus membranaceus*	Anguo, Hebei Province	MT723853
*Papulaspora equi*	*Lycium ruthenicum*	Minqin, Gansu Province	MW548086
*Alternaria tellustris*	*Lycium ruthenicum*	Anxi, Gansu Province	OM936046
*Paraphoma chlamydocopiosa*	*Dendranthema morifolium*	Anguo, Hebei Province	MT723851
*Macrophomina phaseolina*	*Salvia miltiorrhiza*	Yaozhou, Shaanxi Province	OR434038

**Table 2:** Experimental treatment scheme.

Groups	Treatment Group	Methods
Control group	CK	70% field water capacity
Cd stress group	Cd1	5 mg Cd/kg soil
	Cd2	10 mg Cd/kg soil
	Cd3	15 mg Cd/kg soil
Drought stress group	D	40% field water capacity
Drought-Cd interaction stress group	DCd1	40% field water capacity and 5 mg Cd/kg soil
	DCd2	40% field water capacity and 10 mg Cd/kg soil
	DCd3	40% field water capacity and 15 mg Cd/kg soil
Inoculation group	*Np*	Inoculation with *N. phragmitis*
	*At*	Inoculation with *A. tellustris*
Non-inoculation group	*C*	No DSE inoculation

**Table 3:** Fungal TI and IC_50_ to PEG and Cd^2+^ after the incubation of PDA medium exposed to the different tested concentrations for 8 days. IC50 was estimated by fitting the TI data to a logistic model (r^2^ > 0.9). ^a^ 0.80 < r^2^ < 0.9; ^b^ r^2^ ≤ 0.80. Tolerance Index categories: Very low tolerance (0.00–0.39); Low tolerance (0.40–0.59); Moderate tolerance (0.60–0.79); High tolerance (0.80–0.99); Very high tolerance (≥1) [33].

Strain	TI to PEG (%)			IC_50_ to PEG	TI to Cd^2+^ (mg Cd/L)			IC_50_ to Cd^2+^
	9	18	27	(%)	25	50	100	(mg Cd/L)
*Microsphaeropsis cytisi*	0.95	0.72	0.58	30.84 (27.16–38.12)	0.22	0.00	0.00	~22.53
*Alternaria alstroemeriae*	0.99	1.06	0.85	~28.51 ^b^	0.74	0.40	0.27	44.71 (39.98–49.92)
*Stagonosporopsis lupini*	0.94	0.98	0.61	28.36 (~ 30.65)	0.52	0.21	0.00	26.23 (24.19–28.14)
*Neocamarosporium phragmitis*	0.87	1.02	0.57	~27.24 ^a^	0.54	0.39	0.33	29.58 (20.95–36.66)
*Paraphoma chlamydocopiosa*	1.00	1.00	1.00	-	0.48	0.27	0.13	23.51 (22.30–24.66)
*Macrophomina phaseolina*	1.00	1.00	1.00	-	0.43	0.20	0.14	19.25 (15.98–21.97)
*Papulaspora equi*	0.97	0.49	0.00	17.97	0.43	0.22	0.00	22.07 (17.19–25.80)
*Alternaria tellustris*	0.93	0.80	0.60	32.84 (30.34–36.63)	1.01	0.87	0.56	109.30 (99.61–125.00)
*Macrophomina pseudophaseolina*	1.00	1.00	1.00	-	0.43	0.20	0.00	22.08 (19.36–24.35)
*Paraphoma radicina*	1.00	1.00	1.00	-	0.47	0.28	0.11	23.28 (21.77–24.69)

## Data Availability

The original contributions presented in the study are included in the article, further inquiries can be directed to the corresponding authors.
